# Integrated Genome Sequencing and Transcriptome Analysis Identifies Candidate Pathogenicity Genes from *Ustilago crameri*

**DOI:** 10.3390/jof10010082

**Published:** 2024-01-21

**Authors:** Juan Liang, Desuo Yin, Xinyue Shu, Ting Xiang, Chao Zhang, Honglian Li, Aijun Wang

**Affiliations:** 1 College of Plant Protection, Henan Agricultural University, Zhengzhou 450046, China; liangjuan2292@163.com (J.L.); shuxinyuel@163.com (X.S.); xt18728783187@163.com (T.X.); chaozhang21@126.com (C.Z.); honglianli@sina.com (H.L.); 2 College of Agronomy, Sichuan Agricultural University, Chengdu 611130, China; 3 Food Crop Research Institute, Hubei Academy of Agriculture Sciences, Wuhan 430064, China; yindesuo@163.com

**Keywords:** *Ustilago crameri*, foxtail millet, genome, pathogenicity genes, effectors

## Abstract

*Ustilago crameri* is a pathogenic basidiomycete fungus that causes foxtail millet kernel smut (FMKS), a devastating grain disease in most foxtail-millet-growing regions of the world. Here, we report an assembled high-quality genome sequence of *U. crameri* strain SCZ-6 isolated from the diseased grains of foxtail millet in Changzhi, Shanxi Province, China. The genome size is 19.55 Mb, consisting of 73 contigs (N50 = 840,209 bp) with a G + C content of 54.09%, and encoding 6576 predicted genes and 6486 genes supported by RNA-seq. Evolutionarily, *U. crameri* lies close to the barley smut *U. hordei*, and an obvious co-linearity was observed between these two smut fungi. We annotated the genome of *U. crameri* strain SCZ-6 using databases, identifying 1827 pathogen–host interaction (PHI)-associated genes, 1324 genes encoding fungal virulence factors, 259 CAZy-related genes, 80 genes encoding transporters, and 206 putative cytochrome P450 genes; their expression profiles at different inoculation time points were also detected. Additionally, 70 candidate pathogen effectors were identified according to their expression patterns and predicted functions. In summary, our results provide important insights into the pathogenic mechanisms of the pathogenesis-related genes of *U. crameri* and a robust foundation for further investigation.

## 1. Introduction

Foxtail millet kernel smut (FMKS), caused by *Ustilago crameri*, is one of the important grain fungal diseases affecting the production of foxtail millet worldwide [1]. FMKS was first reported in Uttarakhand and then found in India, Karnataka, Andhra Pradesh, Tamil Nadu Maharashtra, and China [1,2,3,4]. FMKS infection occurs at all growth stages of foxtail millet and results in a high incidence of 75% in severe years [1]. A survey of the spread of FMKS in Changzhi, Shanxi Province, China, a major millet-producing area, showed that about 50% of plots investigated were affected by *U. crameri,* with an incidence ranging from 5% to 45% in 2003 and 2004 [5]. FMKS is now an increasing threat to the high production of foxtail millet in most areas where it is grown.

**Figure 1:**
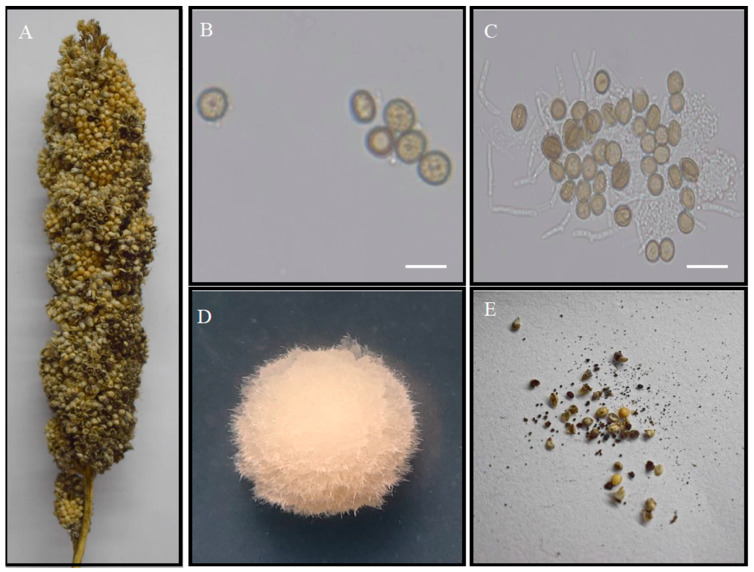
Characteristics of the *Ustilago crameri* strain. (**A**) Hypha infection in foxtail millet kernels and growing points. (**B**) Teliospores scanned using a light microscope. (**C**) Teliospore germination. (**D**) Colony morphology of *U. crameri* after 7 d on PSA. (**E**) Kernel smut balls formed in foxtail millet spikelets. Bars = 100 µm.

*U. crameri* belongs to the Ustilago genus of the Basidiomycota family. A major feature of this pathogen is that it affects grains by producing a dark-black powdery mass of spores in ears (Figure 1A), but sometimes a terminal portion of the spike may escape [6]. Teliospore balls are dark brown and angular or round in shape, measuring 7–10 µm and showing echinulation (Figure 1B). Teliospore balls on seed surfaces can germinate and form promycelia that contribute to primary infections (Figure 1C). The colony morphology of *U. crameri* on artificial media is of the circular type and white (Figure 1D). Hypha infection occurs through the coleoptiles of seedlings and spreads to the ears with foxtail millet growth producing powdery, dark teliospore balls in the kernels during the late phase of infection (Figure 1E). Past studies focused on the epidemic dynamics of and the management practices applied to FMKS [1], while little is known about the pathogenic mechanism of *U. crameri*.

Recently, with the advent of sequencing technologies, combined whole-genome assembled and transcriptome sequencing has become a powerful method for identifying the genes associated with pathogenicity in phytopathogens [7,8]. To date, the pathogenic mechanisms of several smut pathogens, including *U. hordei*, *U. maydis*, *U. scitamineum*, *Sporisorium reilianum*, *Tilletia controversa*, *T. indica*, and *T. horrida*, have been studied using functional genomics and transcriptomics studies [9,10,11]. Wang et al. [11,12], based on de novo and transcriptome-sequencing analysis, revealed that autophagy processes and lipid degradation are key pathogenicity pathways of *T. horrida*. Subsequently, several effectors of *T. horrida*, such as smut_2965, ThSCSP_14, smut_5844, and ThSCSP_12, which trigger necrosis and defense responses in non-host plants, were cloned [13,14,15]. Despite all this, the pathogenic mechanisms of *U. crameri* remain largely unknown, and no effectors have been discovered.

In the present study, we report the high-quality genome sequencing of *U. crameri* SCZ-6, a strain causing FMKS infection in foxtail millet. Furthermore, phylogenetic analyses of *U. crameri* and four other Ustilago pathogens were performed. The results showed that *U. crameri* is closely related to *U. hordei*, whereas the data revealed that 363 genes were present only in *U. crameri*. The candidate pathogenicity genes and effectors of *U. crameri* were also detected through genome annotation and differentially expressed genes (DEGs) analysis. Our results not only provide new insights into the pathogenicity and host specificity of *U. crameri* through a comparison of different species in the genus Ustilago but also establish a database with which to elucidate the possible molecular basis of *U. crameri*–foxtail millet interactions.

## 2. Materials and Methods

### 2.1. Strain Isolates, Culture Conditions, and Genomic DNA and RNA Isolation

We isolated *U. crameri* strain SCZ-6 from symptomatic grains of foxtail millet collected from Changzhi, Shanxi Province, China, in September 2021. The *U. crameri* strain was cultured using a potato dextrose agar (PDA) liquid medium kept at 28 °C for 5 days that was then collected and frozen in liquid nitrogen. A Fungi Genomic DNA Isolation Kit (Sangon Biotech, Inc., Shanghai, China) was used for high-quality genome DNA extraction conducted according to the manufacturer’s protocol.

The foxtail millet cultivar qinzhouhuang (susceptible to FMKS) was inoculated with *U. crameri* strain SCZ-6. The germinated grains were sterilized with 75% alcohol, and then washed for 5 min with sterile water to remove the alcohol and air-dried. Afterward, the germinated grains were put in sterile water, which contained hyphae of *U. crameri.* Then, the mycelium of *U. crameri* that had adhered to the germinated grains was collected at three post-inoculation times (12, 24, and 72 h), immediately frozen in liquid nitrogen, and stored at −80 °C. An Omega Fungal RNA kit was used to isolate the total RNA of *U. crameri*. The total RNA of *U. crameri,* following infection with foxtail millet for 12, 24, and 72 h, was used for transcriptome sequencing. *U. crameri* SCZ-6 mycelia that did not receive any treatment were utilized as controls.

### 2.2. Genome Sequencing and Assembly

The genome DNA sample was fragmented using g-TUBE. The selected fragments were subjected to end repair, adapter ligation, 3′ adenylation, and PCR amplification. After purification with BluePippin, a DNA library was obtained. The qualified libraries were sequenced using the Pacbio Sequel II and Illumina Hiseq 2500 platforms produced by Beijing Biomarker Technology Co., Ltd. (Beijing, China). The PacBio CCC reads were assembled into contigs using Hifiasm [16]. The PacBio assembly results were further corrected for random errors using the IIIumina clean reads via Pilon [17]. The completeness and assembly quality of the SCZ-6 genome were identified through a benchmarking universal single-copy ortholog (BUSCO v2.0) [18].

### 2.3. Gene Prediction and Annotation

Genscan [19], Augustus v2.4 [20], GlimmerHMM v3.0.4 [21], GeneID v1.4 [22], and SNAP (version 2006-07-28) [23] were used for the ab initio gene prediction of *U. crameri* strain SCZ-6. Homologous proteins were detected through GeMoMa v1.3.1 [24]. The assembly used to establish the transcript was performed using Hisat2 v2.0.4 and Stringtie v1.2.3 [25], and the Unigene was predicted using TransDecoder v2.0 [26] and PASA v2.0.2 [27]. The coding genes were generated using PASA v2.0.2 [27] after the predicted data generated with three methods were integrated with EVM v1.1.1 [28]. Furthermore, the repeated sequences were predicted using RepeatMasker v4.0.6 [29]. The transfer RNAs (tRNAs) were predicted using tRNAscan-SE [30], and the ribosomal RNAs (rRNAs), small RNAs (sRNAs), small nuclear RNAs (snRNAs), and microRNAs (miRNAs) were predicted using Infernal 1.1 [31] based on the Rfam database [32].

### 2.4. Gene Function Annotation

The functions of the *U. crameri* protein-encoding genes were annotated using BLAST searches in the Gene Ontology (GO) [33], Kyoto Encyclopedia of Genes and Genomes (KEGG) [34], Clusters of Orthologous Groups (KOG) [35], Non-Redundant Protein (NR) [36], Transporter Classification (TCDB) [37], Pfam [38], and Swiss-Prot database [37] at the threshold of an e-value ≤ 1 × 10^−5^. Pathogenicity-related factors were analyzed based on pathogen–host interactions (PHIs) [39], fungal virulence factors (DFVF) [40], and cytochrome P450 databases [41]. The Carbohydrate-Active Enzyme (CAZy) databases [42] were used for the detection of carbohydrate-active enzymes.

### 2.5. Comparative Genomics Analysis

The genomes of *U. hordei*, *U. maydis*, *U. scitamineum*, and *Sporisorium reilianum* were used for comparison with the genomic data of *U. crameri*. OrthoMCL sofware v1.4 [43] was used to identify the orthologs of the gene family. Genomic synteny was analyzed using MCScanX [44]. PhyML was used for the construction of a phylogenetic tree with 1000 bootstrap replicates [45].

### 2.6. Transcriptome Expression

Beijing Biomarker Technology Co., Ltd. (Beijing, China) constructed a cDNA library of *U. crameri* genome SCZ-6 and performed transcriptome sequencing of the RNA samples. We obtained 125 bp paired-end sequences after carrying out sequencing using the Illumina NovaSeq6000 platform and then removed the low-quality scores or contained adaptor reads from the raw data. The clean reads were aligned to the reference genome *U. crameri* SCZ-6 using HISAT2 [46]. The number of fragments per kilobase of transcript sequence per million (FPKM) of each gene was calculated using StringTie [47], and FPKM values ≥ 1 in at least one of the experimental treatments were considered expressed. The DEGs were analyzed using edgeR [48] with an FDR < 0.05 and a|log2 (fold-change)| > 1.

### 2.7. Secreted Proteins and Potential Effector Analysis

The secretory proteins in *U. crameri* were predicted based on alignment with SignalP 4.0 [49]. The transmembrane helices in the proteins were predicted using TMHMM 2.0 [50]. The proteins with an N-terminal signal peptide (SP) but without transmembrane helices, which induced upregulation after inoculation, were predicted to be potential effector proteins [14].

### 2.8. Quantitative Real-Time Reverse Transcription–Polymerase Chain Reaction

The RNA samples from transcriptome sequencing were used for quantitative real-time reverse transcription–polymerase chain reaction (qRT-PCR). The qRT-PCR was conducted using the Bio-Rad CFX96 Real-Time PCR System (Bio-Rad, Foster City, CA, USA) to observe the relative expression levels of the selected genes. The fungal conserved gene *UBQ* was used as an internal reference gene to determine the values of the relative expression levels. The relative expression levels of the selected genes were calculated using the 2^–ΔΔCt^ algorithm. Four biological replicates were used. Statistical analysis was conducted using a one-way analysis of variance, followed by a Tukey’s multiple comparison test. The primers used for this study are provided in Supplementary Table S1.

## 3. Results

### 3.1. Genome Sequencing and Assembly

The genome of *U. crameri* strain SCZ-6 was sequenced using the Pacbio Sequel II and Illumina Hiseq 2500 platforms provided by Beijing Biomarker Technology Co., Ltd. (Beijing, China). In total, 147,035 PacBio Circular Consensus Sequencing (CCS) reads with 0.998 Gb of filtered subread bases and a total read length of 742,113,011 bp were generated using PacBio Sequel II sequencing (Supplementary Table S2 and Supplementary Figure S1). The average length of the CCS was 7730.398 bp, and the maximum length was 24,648 bp (Supplementary Figure S1). Hifiasm [16] was used for the de novo assembly of the clean reads, and Pilon [17] was used to correct random errors in the raw assembly sequences. The final genome assembly size of *U. crameri* isolate SCZ-6 was 19.55 Mb, consisting of 73 contigs with an N50 of 840 and 209 bp with a GC content of 54.09% (Table 1). Furthermore, the assessment of genome completeness showed that 282 (97.24%) complete BUSCOs were found and that the genome was well assembled (Supplementary Table S3).

### 3.2. Genome Annotation

Ab initio gene prediction, RNA sequencing data prediction, and homologous protein prediction were used to identify functional genes. The predicted results yielded by the above three methods were integrated using EVidenceModeler (EVM) v1.1.1 [28]. Among the annotated 6576 protein-coding genes, 6486 protein-coding genes were supported by the RNA-seq data and homologous protein prediction (Supplementary Figure S2). The predicted genes constituted 61.22% of the assembled genome, with an average length of 2308.48 bp (Supplementary Table S4). The predicted genes were annotated using the NCBI nonredundant protein (6478 genes), Swiss-Prot (4514 genes) (Bairoch and Apweiler, 2000), protein family (5180 genes) [51], gene ontology (GO) (4872 genes) [33], TrEMBL(6476 genes) [52], clusters of orthologous groups for eukaryotic complete genomes (4122 genes) [35], and Kyoto Encyclopedia of Genes and Genomes (KEGG) (2802 genes) [53] (E-value < 1 × 10^−5^) databases. Furthermore, 457 noncoding RNAs, including 34 ribosomal RNAs (rRNAs), 357 transfer RNAs (tRNAs), and 66 others noncoding RNAs (Supplementary Table S5), were detected using tRNAscan-SE, Infernal V1.1, and the Rfam data bank [31,54,55]. In total, 338 (3.72%) repetitive sequences were found in the *Ustilago crameri* SCZ-6 genome (Supplementary Table S6). Among the repetitive elements, the potential host gene was the largest part of the repetitive sequences and accounted for 2.76% (Supplementary Table S6).

### 3.3. Comparative Genomics of Five Smut Fungi

**Figure 2:**
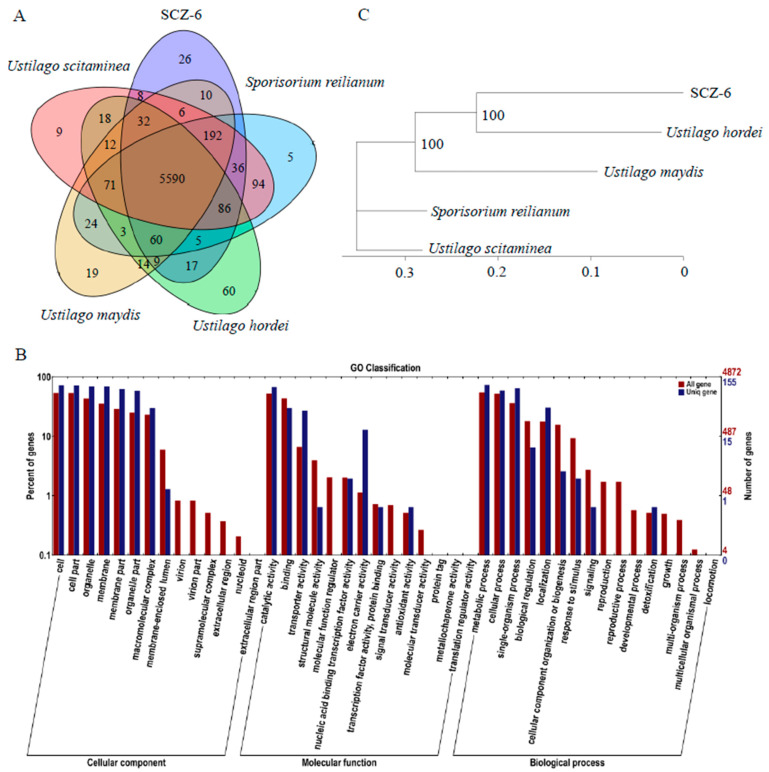
Phylogenetic relationship between *Ustilago crameri* and other smut fungi. (**A**) Venn diagram showing orthologs between the five sequenced smut fungi. The values explain the counts of ortholog groups and the counts of genes in parentheses. (**B**) The 363 predicted genes that appeared to be unique to *U. crameri* SCZ-6 according to Gene Ontology (GO) annotation. (**C**) The phylogeny of 5 smut fungi. Phylogeny was constructed using Mega 7 with single-copy genes. Protein alignments were analyzed using MUSCLE3.8.31.

Four other smut fungi that infect different hosts were used for the comparative genomics. The gene families of *U. crameri* and four smut fungi were evaluated using OrthoMCL. This analysis revealed that the *U. crameri* genome consisted of 6127 gene families, and 5590 gene families were co-existing in all five smut fungi; however, a total of 26 gene families (containing 363 predicted genes) were only found in *U. crameri* SCZ-6 (Figure 2A). A GO enrichment analysis of these 363 unique genes was further performed, and the results showed that transporter activity and electron carrier activity were significantly enriched compared to all the other genes (Figure 2B). We speculated that these GO terms may play crucial roles in the *U. crameri*–host interaction that need to be explored.

Additionally, phylogenetic analysis revealed a high similarity between *U. crameri*, *U. hordei*, *U. maydis*, *U. scitamineum*, and *Sporisorium reilianum*; however, *U. crameri* was more phylogenetically related to *U. hordei* than to *U. maydis*, *U. scitamineum*, and *S. reilianum* (Figure 2C). Synteny analyses between *U. crameri* and *U. hordei*, *U. maydis*, *U. scitamineum*, and *S. reilianum* were further conducted. The results showed that *U. crameri* had a close relationship with *U. hordei*, sharing 8325 synteny blocks in the 16.8 Mb region, accounting for 85.93% of the *U. crameri* SCZ-6 genome (Supplementary Figure S3). We also found that there are 6780 (accounting for 79.79% of the *U. crameri* SCZ-6 genome), 6576 (77.74%), and 6576 (77.74%) synteny blocks between the genomes of *U. crameri* and *U. maydis*, *U. scitamineum*, and *S. reilianum*, respectively (Supplementary Figure S3). On the other hand, the clustering of four different smut pathogens, *U. crameri*, *U. hordei*, *U. maydis*, and *U. scitamineum*, belonging to the genus Ustilago, onto four distinct branches suggests the varying infectious capabilities of these four pathogens toward different hosts within the Gramineae family, implying their coevolutionary dynamics with their respective hosts.

### 3.4. Transcriptome Analysis during Infection

**Figure 3:**
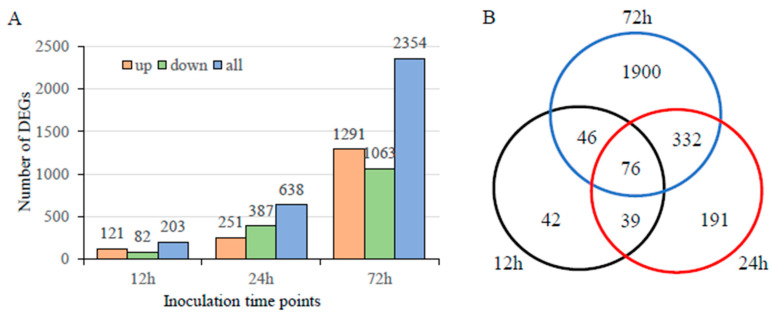

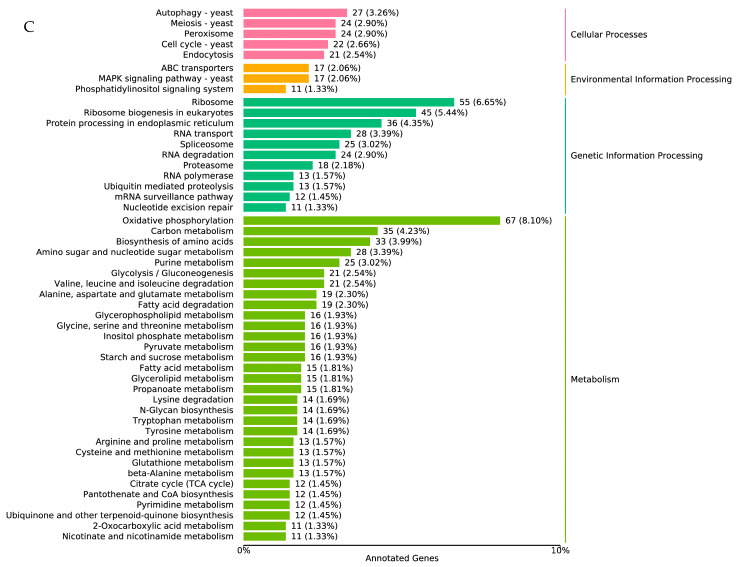
Differentially expressed genes (DEGs) in *U. crameri* SCZ-6 after inoculation. (**A**) Number of upregulated and downregulated DEGs in *U. crameri* SCZ-6 after inoculation. (**B**) Venn diagrams showing the overlapping of the DEG numbers in *U. crameri* SCZ-6 at different inoculation time points (12, 24, and 72 h). (**C**) The 3241 DEGs in *U. crameri* SCZ-6 according to Kyoto Encyclopedia of Genes and Genomes (KEGG) annotation.

To elucidate the important disease-causing genes expressed during the infection process, we investigated the transcriptomes of *U. crameri* at three inoculation time points (12, 24, and 72 h). Compared with the uninfected strains, there were 121, 251, and 1291 genes significantly upregulated at 12, 24, and 72 h post-inoculation (hpi); however, 82, 387, and 1063 genes were significantly downregulated at 12, 24, and 72 hpi (FDR < 0.05 and |log2 Fold Change| > 1; Figure 3A). Among these differentially expressed genes (DEGs), 76 DEGs were shared by three time points; 42, 191, and 1900 DEGs were uniquely detected in 12, 24, and 72 h (Figure 3B). Furthermore, the functions of these DEGs at the three time points were annotated based on the KEGG database, and we found several genes involved in autophagy, the MAPK signaling pathway, fatty acid degradation, and glutathione metabolism (Figure 3C); these pathways are closely related to the pathogenicity of a phytopathogen [56]. Thus, our results provide useful information for detecting the genes involved in the pathogenesis of *U. crameri*.

### 3.5. Carbohydrate-Active Enzymes

**Figure 4:**
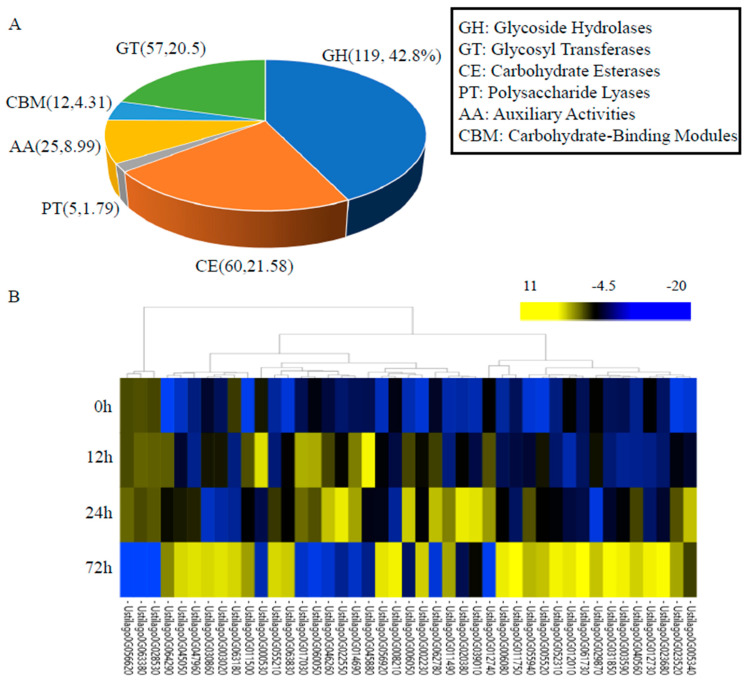
The carbohydrate degradative enzyme (CAZyme)-coding genes in the *U. crameri* SCZ–6 genome. (**A**) The proportion (%) of different types of CAZymes in the *U. crameri* SCZ–6 genome. (**B**) The expression patterns of 52 upregulated genes encoding the CAZymes of *U. crameri*.

Plant pathogenic fungi can secrete some CAZymes to degrade host cell walls, resulting in the promotion of pathogen infection [42]. Thus, a database pertaining to CAZymes was further used for gene functional annotation. There were 259 genes that were annotated as CAZymes, which included 119 glycoside hydrolases (GHs), five polysaccharide lyases (PLs), 60 carbohydrate esterases (CEs), 57 glycosyl transferases (GTs), 25 auxiliary activities (AAs), and 12 carbohydrate-binding modules (CBMs) (Figure 4A). Among these 259 genes encoding CAZymes, 52 were upregulated after *U. crameri* infection; their expression patterns over the three infection stages are shown in Figure 4B. Ustilago0G045880, which encoded PGU1-Endo-polygalacturonase, was upregulated at 12 h after infection (Figure 4B); along with polygalacturonase, they are both important pathogenicity factors of phytopathogenic fungi [57]. Similarly, several genes that encoded glucosidase, such as Ustilago0G014690, Ustilago0G064290, and Ustilago0G020380, were also upregulated at 12 h after infection (Figure 4B). Thus, we speculate that these genes may play crucial roles in the *U. crameri*–host interaction during early infection.

### 3.6. Important Genes Involved in Pathogenicity

**Figure 5:**
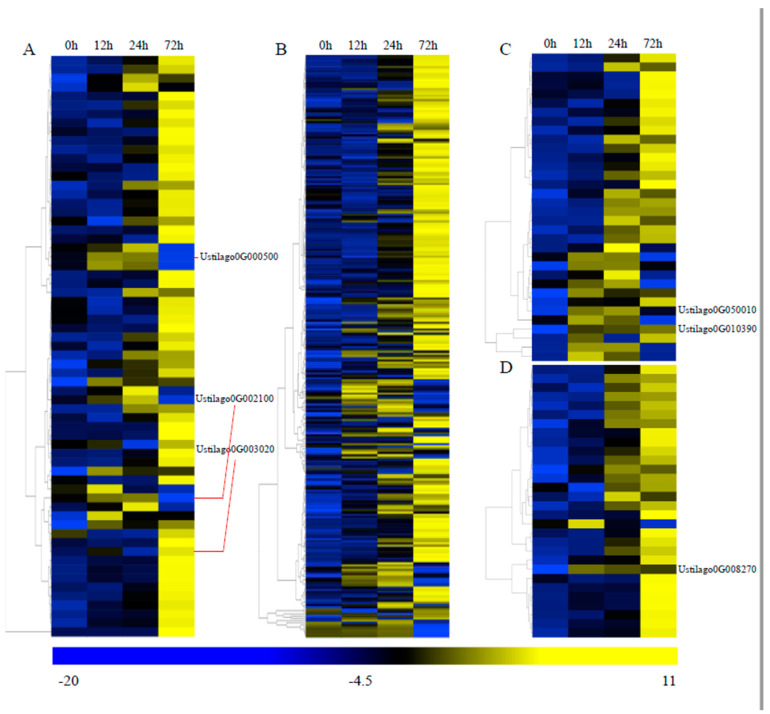
The expression patterns of upregulated pathogen–host interaction (PHI) −based genes and genes for fungal virulence factors (FVFs), cytochrome P450s (CYP450s), and transporters during *U. crameri* infection. (**A**) A total of 65 genes involved in PHIs were upregulated at 12, 24, and 72 h post–inoculation (hpi). (**B**) A total of 264 genes involved in FVFs were upregulated at 12, 24, and 72 hpi. (**C**) A total of 34 genes involved in CYP450s were upregulated at 12, 24, and 72 hpi. (**D**) A total of 30 genes involved in transporters were upregulated at 12, 24, and 72 hpi.

To further detect the pathogenicity-related genes, the pathogen–host interaction (PHI) database was used for a search against the *U. crameri* genome. We found 1827 putative PHI genes in the *U. crameri* genome. The expression profiles of 1827 putative PHI genes at three infection stages (12, 24, and 72 h) were observed. Among these 1827 putative PHI genes, 124 DEGs (65 upregulated; 59 downregulated) were detected (Supplementary Table S7). The expression of the 65 upregulated DEGs is shown in Figure 5A. Ustilago0G003020, which encodes a protein related to chitinase that is associated with cell wall degradation [58], exhibited induced upregulation at 12 h. In addition, many fungi can degrade salicylic acid (SA) into catechol in the fungal cytosol by producing salicylate hydroxylases, which can decrease the SA levels in an infected host [59]. We found that the salicylate-hydroxylase-encoding gene Ustilago0G002100 had upregulated expression at 12 h. There is evidence showing that some phytopathogenic fungi can suppress host defenses mediated by reactive oxygen species (ROS) through synthesizing mannitol [60]. Mannitol accumulation is accompanied by the high expression of a mannitol dehydrogenase (MAD1) in haustoria [60]. The mannitol dehydrogenase encoded gene Ustilago0G000500 also exhibited induced upregulation at 12 h. Thus, we assumed that the expression of these genes might play an important role in the interaction between *U. crameri* and host plants.

We also used the fungal virulence factors (DFVF, v6) database to identify the pathogenicity-related genes, and 1324 genes encoding fungal virulence factors were annotated. Among these 1324 genes, 264 were upregulated DEGs (Figure 5B; Supplementary Table S8), indicating the crucial role of these fungal virulence factors in the pathogenicity of *U. crameri*. Furthermore, cytochrome P450s (CYP450s) are related to the production of fungal toxins [61,62]; for example, the *Aspergillus parasiticus* CYP450 gene cypX is involved in aflatoxin biosynthesis [63]. Additionally, transporters are not only related to the secretion of toxins but also participate in the acquisition of carbon and nitrogen sources from their host plants [64]. Thus, the pathogenesis-related genes were also searched against the transporter classification database (TCDB) and the CYP450s engineering database (CYPED). In total, 80 genes encoding transporters and 206 putative cytochrome P450 genes were found. Among the 34 upregulated DEGs encoding CYP450s (Figure 5C; Supplementary Table S9), we found the major facilitator superfamily (MFS) transporters Ustilago0G050010 and Ustilago0G010390 exhibited induced upregulation at 8 h, and MFS transporters are essential to the ability of phytopathogenic fungi to deal with SA stress from their hosts [65]. Additionally, among the 30 upregulated transporters (Supplementary Table S10), only the ENA2-Plasma membrane P-type ATPase encoded gene Ustilago0G008270 was continuously induced at the three inoculation time points (Figure 5D). These genes might be important determinants of *U. crameri* virulence.

### 3.7. U. crameri Candidate Effectors

**Figure 6:**
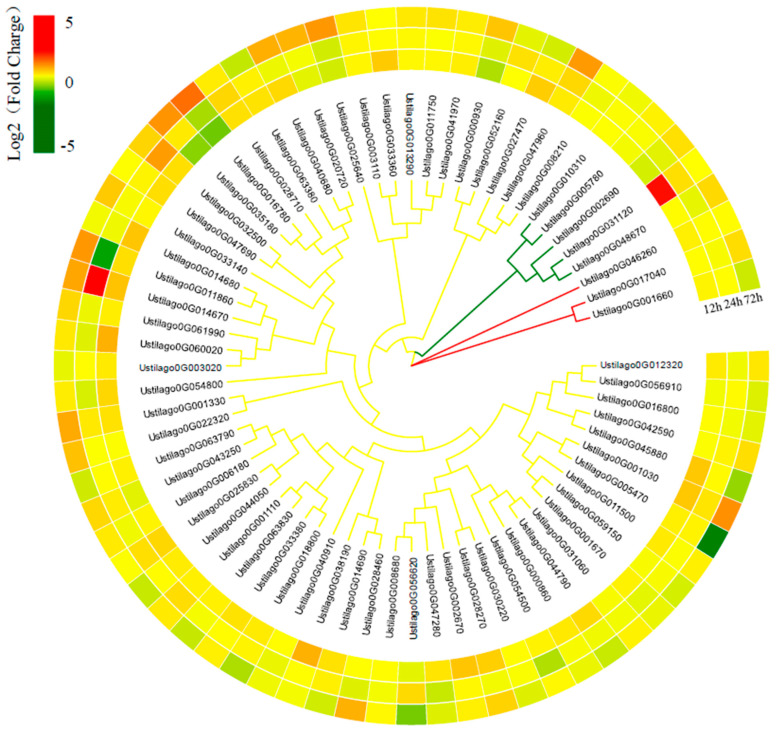
The phylogeny and expression of 70 candidate effectors in the genome of *U. crameri* SCZ–6.

The SignalP v4.0 [66] prediction demonstrated that 543 proteins (8.26%) contained signal peptides. Among them, 228 proteins contained predicted transmembrane helices, and another 315 proteins without transmembranes were predicted to be secreted proteins (Table 1). Additionally, based on the analysis of the RNA-seq data, we identified 70 upregulated secreted protein encoded genes as potential *U. crameri* effectors (Figure 6; Supplementary Table S11). We confirmed the expression of several predicted effector genes using RT-qPCR (Supplementary Figure S4). A phylogenomic analysis of these candidate effectors classified them into three clusters (Figure 6). Among these 70 candidate effectors, some candidate effectors with conserved functional domains such as glycoside hydrolase, Herpes_BLLF1, CuRO_3_Fet3p, DPBB_RlpA_EXP_N-like, and Ribosomal_L11 domains might play significant roles in fungal pathogenesis (Supplementary Table S11). In addition, we also found the homologous gene of UhAvr1 (a key effector in *U. hordei*) [67], Ustilago0G035610, whose genomic loci are 420,639–421,753 bp, and it did not show synteny with UhAvr1 in the *U. hordei* genome (Supplementary Figure S3B).

## 4. Discussion

*U. crameri* is a devastating pathogen that influences foxtail millet production. In this study, the draft genome of *U. crameri* strain SCZ-6, which was isolated from Changzhi, Shanxi Province, China, was sequenced and assembled. *U. crameri* SCZ-6 has a genome size of 19.55 Mb, which is closer in size to the genomes of *U. maydis* (19.66 Mb) and *S. scitamineum* (19.42 Mb) than the other genus smut fungal genomes [11]. Furthermore, *U. crameri* SCZ-6 has a larger genome size than *U. crameri* strain Uc7 (18.82 Mb), which was isolated from Chengde, Hebei Province, China [68]. Importantly, the expression patterns of pathogenicity genes in *U. crameri* SCZ-6 at different inoculation times were clarified through transcriptomic analysis; however, we only completed sequencing and simple annotation for the genome of *U. crameri* Uc7 [68]. Thus, our results will supply more detailed information for the study of the interaction between *U. crameri* and its host.

Previous research has shown that biotrophic fungal pathogens contain fewer CAZyme encoded genes than hemi-biotrophic and necrotrophic pathogens [69]. Biotrophic fungal pathogens can minimize the release of cell wall fragments through producing fewer carbohydrate-active enzymes, whose products are often recognized as endogenous signals to induce plant immunity [70,71]. Our results show that *U. crameri* has 259 genes that were annotated as CAZymes, accounting for 3.94% of the predicted genes in the *U. crameri* genome. Interestingly, we found that the upregulation of 52 CAZymes was induced by *U. crameri* infection, and these CAZyme encoded genes may play a unique role in the pathogenicity of *U. crameri.* Further studies are needed to explore the virulence mechanisms of these CAZymes in *U. crameri* and advance our understanding of the pathogenic pathways involved in the foxtail millet–*U. crameri* interaction.

A correlativity study showed that the MFS transporters of plant pathogens are mainly associated with nutrient uptake and antifungal drug resistance [72]. The MFS transporters TRI12 from the maize pathogen *Fusarium sporotrichioides* [73] and CFP from the soybean pathogen *Cercospora kikuchii* [74] are needed for the secretion of the fungal toxins trichothecenes and cercosporin, respectively. Chen et al. [66] showed that the MFS transporter FgMFS1 was highly expressed during *Fusarium graminearum* infection and plays a critical role in the response to wheat endogenous SA and pathogenicity toward wheat. Among 34 upregulated DEGs encoding CYP450s, we found two MFS transporters, Ustilago0G050010 and Ustilago0G010390, that were strongly induced during *U. crameri* strain SCZ-6 infection. These results suggest that Ustilago0G050010 and Ustilago0G010390 are important for the pathogenicity of *U. crameri* strain SCZ-6.

Effectors play an essential role in the infection of hosts by a phytopathogen [75]. In recent years, many pathogenic effectors of pathogens have been cloned. For species of smut fungi, the effectors Pit2, See1, Pep1, Cmu1, and Tin2 in the *U. maydis* genome have been studied [76,77,78,79,80]. Among them, the effector See1 plays a significant role in the formation of leaf tumors in maize [77], and Pit2 can promote the infection of *U. maydis* through inhibiting the activity of host cellular proteases [76]. We obtained 315 putative secreted proteins from the *U. maydis* SCZ-6 genome. In general, the genes encoding effector proteins exhibited induced upregulation in the process of infecting a plant pathogen [81]. Thus, the 70 upregulated secreted proteins were considered potential *U. maydis* effectors. Our research provides important gene information for the study of the pathogenic mechanism *U. crameri* and can help build a bridge promoting the comprehension of the interaction mechanism between *U. crameri* and its host.

## 5. Conclusions

Overall, we assembled the high-quality genome of the causal agent of foxtail millet kernel smut, *U. crameri* SCZ-6, using PacBio and the Illumina sequencing method. A functional annotation of *U. crameri* genes was also conducted, helping us to further detect the pathogenic mechanism of *U. crameri* at the genome level. We further predicted the CAZymes, PHI genes, and effectors, and these genes are key virulence factors that can promote *U. crameri* infection in foxtail millet. Our results are crucial for further disease-related functional gene studies on *U. crameri* as well as for the FMKS resistance breeding of foxtail millet.

## Figures and Tables

**Table 1:** Genome features of *Ustilago crameri* SCZ-6.

Genomic Features	Numbers
Genome size (Mb)	19.55
Coverage	151.06×
Number of contigs	73
N50 (bp)	840,209
N90 (bp)	396,038
GC content (%)	54.09
Repeat rate (%)	3.72
Predicted protein-coding genes	6576
Average gene length (bp)	2308.48
Exons number	10,145
Average exon length (bp)	1438.95
Introns number	3569
Average intron length (bp)	163.19
tRNA	357

## Data Availability

The draft genome sequence has been deposited in the NCBI Sequence Read Archive under BioProject PRJNA947366 and Biosample SAMN33848936.

## Supplementary Materials

The following supporting information can be downloaded at https://www.mdpi.com/article/10.3390/jof10010082/s1, Supplementary Figure S1: The CCS reads length distribution of *Ustilago crameri* strain SCZ-6, Supplementary Figure S2: The number of protein-coding genes predicted by ab initio gene prediction, RNA sequencing data prediction, and homologous proteins prediction, respectively, Supplementary Figure S3: Genome assembly sequence comparisons between *Ustilago crameri* and *Sporisorium reilianum* (A), *U. hordei* (B), *U. maydis* (C), and *U. scitaminea* (D), Supplementary Figure S4: Expression analysis of six predicted effector genes at different inoculation time points by qRT–PCR. Statistical analysis was performed using a one-way ANOVA, followed by a Tukey’s multiple comparison test. Error bars are the standard deviation (SD) of four independent replicates (ns represents no significant difference; * *p* < 0.05; ** *p* < 0.01; *** *p* < 0.001), Supplementary Table S1: The primer sequences used in this study, Supplementary Table S2: The statistics of CCS reads, Supplementary Table S3: The statistics of the BUSCO assessment, Supplementary Table S4: The statistics of protein-coding genes, Supplementary Table S5: The statistics of noncoding RNAs, Supplementary Table S6: The statistics of repetitive sequences, Supplementary Table S7: The predicted PHI DEGs, Supplementary Table S8: The predicted DFVF upregulated DEGs, Supplementary Table S9: The predicted CYP450 upregulated DEGs, Supplementary Table S10: The predicted transporters upregulated DEGs, Supplementary Table S11: The expression and conserved domains of 70 candidate effectors.
