# Supplementary material for: Integrated Genome Sequencing and Transcriptome Analysis Identifies Candidate Pathogenicity Genes from Ustilago crameri
Source: J Fungi (Basel). 2024 Jan 21;10(1):82. doi: 10.3390/jof10010082 (PMC10821473; doi:10.3390/jof10010082)
Supplement: Supplementary file 1 [file jof-10-00082-s001.zip › Supplementary Figure.pdf]

Integrated genome sequencing and transcriptome analysis identifies candidate pathogenicity genes from *Ustilago crameri*

Juan Liang [1,2,†], Desuo Yin [3,†], Xinyue Shu [1,2], Ting Xiang [1,2], Chao Zhang [1], Honglian Li [1] and Aijun Wang [1,*]

[1] College of Plant Protection, Henan Agricultural University, Zhengzhou 450046, China
[2] College of Agronomy, Sichuan Agricultural University, Chengdu 611130, China
[3] Food Crop Research Institute, Hubei Academy of Agriculture Sciences, Wuhan 430064, China
* Correspondence: ajwang6174@126.com
† These authors contributed equally to this work.

Supplementary Figures

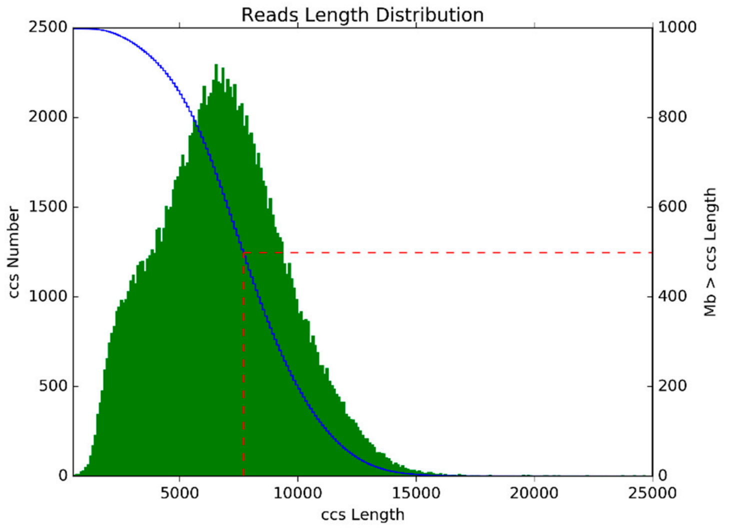

Supplementary Figure S1 The CCS reads length distribution of *Ustilago crameri* strain SCZ-6.

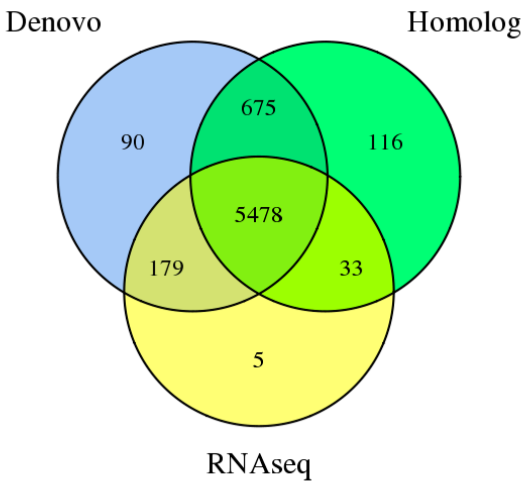

Supplementary Figure S2 The number of protein-coding genes predicted by ab initio gene prediction, RNA sequencing data prediction, and homologous proteins prediction, respectively.

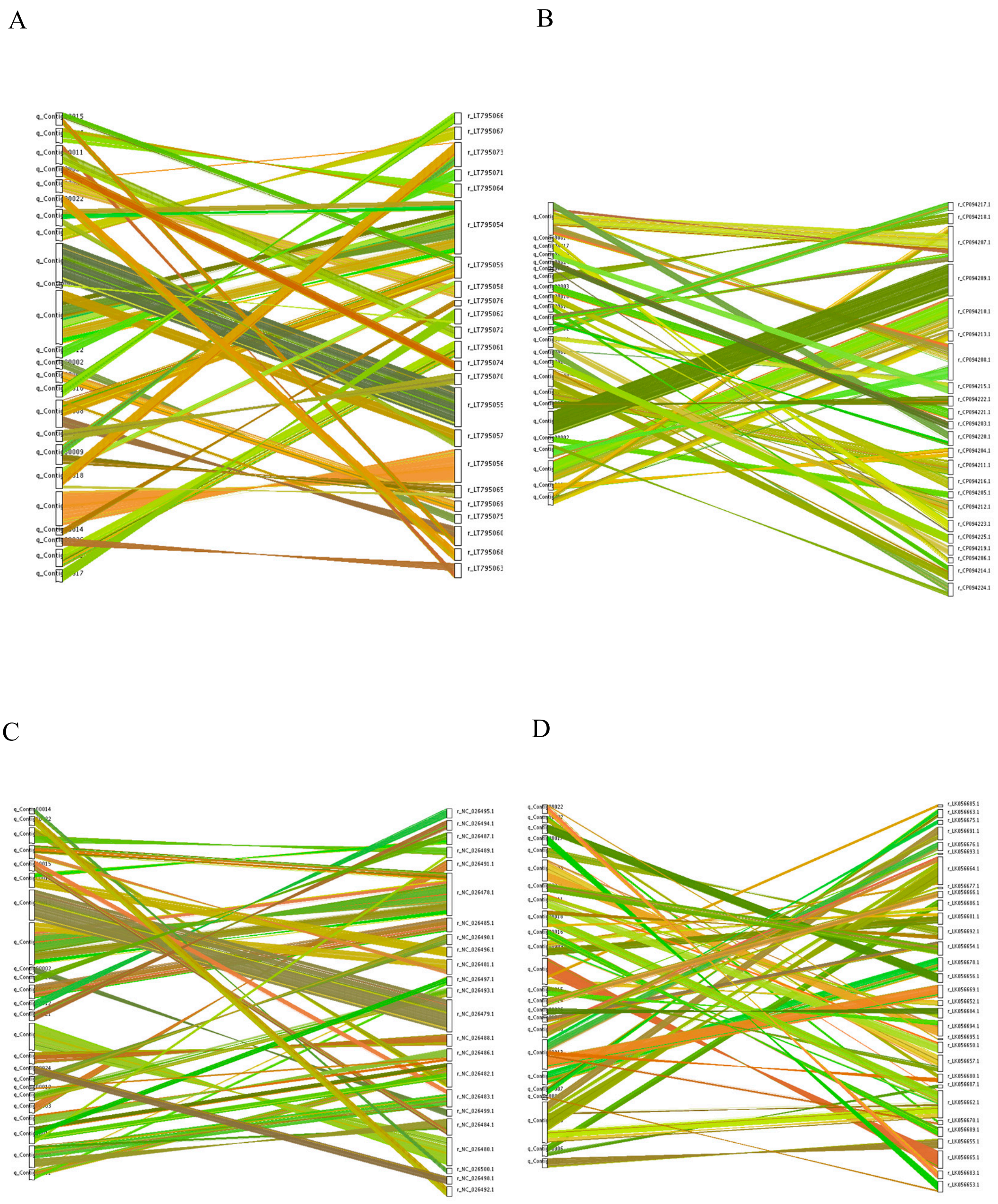

Supplementary Figure S3 Genome assembly sequence comparisons between *Ustilago crameri* and *Sporisorium reilianum* (A), *U. hordei* (B), *U. maydis* (C), and *U. scitaminea* (D).

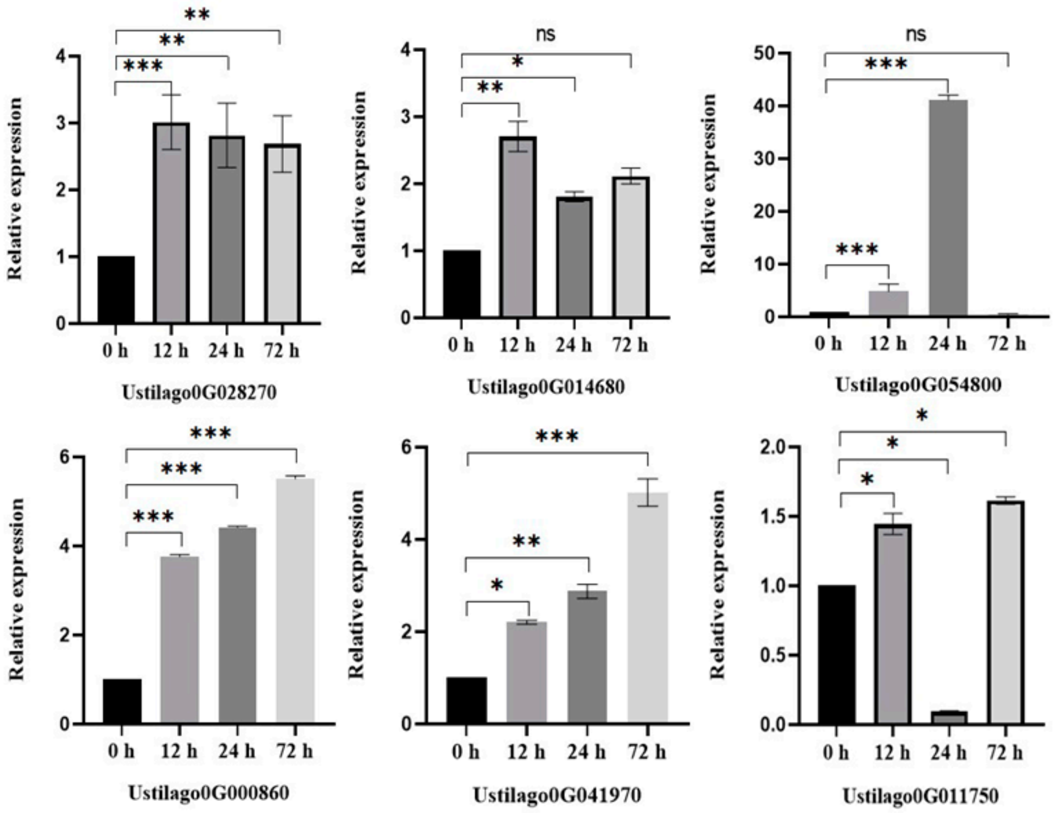

Supplementary Figure S4 Expression analysis of six predicted effector genes at different inoculation time points by qRT–PCR. Statistical analysis was performed by one-way ANOVA, followed by Tukey's multiple comparison test. Error bars are the standard deviation (SD) of four independent replicates (ns represent no significant difference; *P<0.05; **P<0.01; *** P < 0.001).
